# 781 Visual Representation of Race and Skin Tones in North American Burn Literature

**DOI:** 10.1093/jbcr/irae036.322

**Published:** 2024-04-17

**Authors:** Cameron J Kneib, Ryann E Shor, Tam N Pham, Barclay T Stewart

**Affiliations:** UW Medicine Regional Burn Center, Seattle, WA; University of Washington, Harborview Burn Centre, Seattle, WA; University of Washington, Seattle, WA; UW Medicine Regional Burn Center, Seattle, WA; University of Washington, Harborview Burn Centre, Seattle, WA; University of Washington, Seattle, WA; UW Medicine Regional Burn Center, Seattle, WA; University of Washington, Harborview Burn Centre, Seattle, WA; University of Washington, Seattle, WA; UW Medicine Regional Burn Center, Seattle, WA; University of Washington, Harborview Burn Centre, Seattle, WA; University of Washington, Seattle, WA

## Abstract

**Introduction:**

Representation of all people in the medical literature enhances our collective ability to practice health equity and avoid implicit bias in patient evaluation, treatment, and outcomes determination. Clinical photographs play an important role in evaluation of burn care and its outcomes. Prior research has shown disparities of photographic representation of minorities in surgical literature. Our aim was to sample diversity of skin color in images from burn journals to represent diversity of patients in the current burn literature from high-income North American countries (NA).

**Methods:**

A bibliometric analysis was performed on three leading burn surgery journals between 2021-2023. Each report was reviewed by two independent reviewers to evaluate medical images depicting native human skin and location of corresponding author. Images and number of distinct individuals (for figures containing multiple photos of the same individual) were tabulated and categorized as Fitzpatrick Scale ◻3 (White/fair skin tone) or >3 (non-White/dark skin tone).

**Results:**

A total of 353 articles in the Journal of Burn Care and Research, Burns, European Burn Journal with a corresponding author based in NA were reviewed for the study period, of which 45 (13%) articles contained images of native human skin. Analysis showed 216 photos depicting White/fair skin tone (60%) in 52 individuals (52%), whereas 144 photos depicted non-White/dark skin tone (40%) in 35 individuals (35%). This proportion is comparable to published American Burn Association factsheets for incidence of burns in non-White/non-Caucasian (41%).

**Conclusions:**

Current images of non-White race/skin tone in burn literature from high-income North American countries indicate proportional race representation compared to NA burn injury demographics. Thoughtful and more intentional use of photographs depicting diverse skin tones improves representation within the field of burn care.

**Applicability of Research to Practice:**

Greater awareness and intentional use of diverse skin tones in publication photographs are important for equity.

